# Doffing procedures of personal protective equipment evaluated with lipid nanoparticles as viral surrogates: uncovering potential blind spots

**DOI:** 10.1186/s13756-025-01680-w

**Published:** 2025-12-11

**Authors:** Lara Pfuderer, Andrée Friedl, Benedikt Wiggli, Robert Grass

**Affiliations:** 1 Department of Chemistry and Applied Biosciences, Institute for Chemical- and Bioengineering, ETH Zurich, Vladimir-Prelog-Weg 1-5/10, 8093 Zurich, Switzerland; 2 Infectious Diseases & Infection Prevention, Cantonal Hospital of Baden, Baden, Switzerland

**Keywords:** Disinfection, Doffing procedures, Lipid nanoparticles, Viral surrogates; personal protective equipment

## Abstract

**Background:**

Personal protective equipment (PPE) should effectively protect health care workers (HCWs) when treating infectious patients. However, during doffing contamination from outside of the PPE could be transferred and might cause serious infection. Therefore, complex doffing procedures have been developed, which include disinfection steps and would thereby protect the HCWs even if a contamination event occurred during doffing. However, assessing these complex multi-step procedures regarding risk of contamination and infection is challenging. The use of harmless surrogates with pathogen mimicking properties such as lipid nanoparticles encapsulating DNA (LNPs) could provide valuable insights into the effectiveness of doffing and disinfection procedures. Compared to the state-of-the-art method of contamination monitoring using fluorescent lotions LNPs promise to be more sensitive and give additional insights into the value of the disinfection steps.

**Methods:**

After pre-testing the suitability of LNPs as viral surrogates in terms of detection limit and susceptibility to ethanolic disinfection, LNPs with different barcodes were used to evaluate the PPE doffing procedure in place at the Cantonal Hospital Baden (Switzerland). During the biannual HCWs’ PPE training, several sites of the PPE were deliberately contaminated with LNPs after donning. After completion of the doffing procedure, the hands and faces of the HCWs and several environmental sites were analysed for LNP contamination via qPCR.

**Results:**

The analysis showed that no contamination of HCWs’ hands and faces was detectable, indicating the effective protection of HCWs. But some environmental sites were contaminated during the doffing procedure. Owing to the disinfection sensitivity of the LNPs it could be shown that the LNPs detected were disintegrated during one of the disinfection steps of the procedure.

**Conclusions:**

This study demonstrates that LNPs can be used as viral surrogates during the evaluation of PPE doffing procedures. LNPs can lead to insightful results due to their low detection limit and the susceptibility towards disinfection, making this method superior to fluorescent lotions. Consequently, indications for the procedures’ effectivity in inhibiting pathogen transfer to HCWs were found using LNPs. At the same time, blind spots in environmental contamination were uncovered, and the necessity of the disinfection steps in the protocol was displayed.

**Supplementary Information:**

The online version contains supplementary material available at 10.1186/s13756-025-01680-w.

## Background

When treating infectious patients the safety of the healthcare personnel is of the utmost importance. Personal protective equipment (PPE)—such as gloves, face masks, aprons, and goggles—are used to prevent the transfer of pathogens to the healthcare worker (HCW) [1]. Particularly, with highly infectious and deadly viruses, such as Ebola, effective use of PPE is critical [2]. Self-contamination of the HCW can occur during doffing and can potentially lead to transfer of the infectious pathogen from the PPE to the HCW [3]. To exclude self-contamination procedures typically include several levels of redundancy (e.g. multiple layers of gloves), as well as surface disinfection steps, which aim at inactivating pathogens in case of a contamination event. However, it is difficult to control HCW in doffing PPE because the protocols are complex and involve many steps. Therefore, potentially hazardous transfer events may be overlooked. In order to assess the effectiveness of the doffing protocol and provide feedback and reassurance to the HCW, several studies have been conducted. To test the doffing procedure of PPE camera surveillance [4] and fluorescent lotion [3, 5] have previously been used to identify contamination events. However, in both cases the analysis was performed by image analysis and is therefore subjective. In addition, the methods have relatively high detection limits and are not able to detect minute contamination events, which might be relevant when working with highly infective pathogens. Another option for testing PPE doffing procedures are surrogates modelling the pathogen’s properties. For example, bacteriophages have been previously used as viral surrogates [6, 7] offering much lower detection limits than the imaging methods. In addition, bacteriophages can be used to assess the function and efficiency of disinfection steps, but this analysis of viable phages via plaque assay is time-consuming [8, 9]. In order to successfully track multiple contamination sites simultaneously, multiple analytically distinguishable units of the surrogate, called barcodes, can be used. However, the availability of barcodes of bacteriophages is limited to the natural occurring variants [6]. Lipid nanoparticles encapsulating DNA (LNP) have been proposed as microbial surrogates since they can also be used to assess disinfection steps due to their lipid membrane and the encapsulated synthetic DNA allows for an unlimited number of barcodes and a very low detection limits via qPCR [10–12]. These low detection limits are particularly insightful if LNPs are used to investigate contamination events with pathogens, which can be infectious at very low doses, such as the Ebola virus, where 10 or fewer particles can still cause infectious [13].

The aim of this study is to evaluate LNPs regarding their suitability as viral surrogates for the investigation of PPE doffing procedures. For this, pre-experiments were carried out to characterise LNPs by their detection limit and their ability to discriminate contamination and disinfection events, which are analytically accessible as LNP transfer and LNP integrity measures. The usefulness of these measures was then contrasted with the performance of the commonly applied method for testing PPE doffing procedure, namely fluorescent lotion [1, 6]. Consequently the PPE doffing procedures for strict isolation in place at the Cantonal Hospital Baden (Aargau, Switzerland) were evaluated using LNPs.

## Methods

### LNP formulation, sampling and analysis

LNPs using SM-102 as the ionisable lipid were prepared as described elsewhere [10]. In brief, three different LNP barcodes were prepared, meaning LNPs that each encapsulate a different DNA sequence serving as unique identifiers. The final LNP formulation used comprised of 100 µl LNP (2 g/l), 300 µl external DNA (1.6 g/l, salmon sperm DNA salt, Sigma-Fine Chemicals), 300 µl glycerine (100%) and 600 µl sucrose (saturated solution in milliQ water). Per contamination spot 100 µl LNP formulation was applied using a pipette. To evaluate whether surfaces or participants were contaminated with LNPs, samples were taken from the surface by rolling a swab (PCR-Nasal swab, MFS-96000BQ, Meidke Gene), premoistened with milliQ water, over the sampled surface. The swab was then placed in a clean Eppendorf tube and cut to fit within the tube. Analysis was performed as described elsewhere [10]. In general, two parameters can be measured via qPCR: First, overall LNP concentration (via measuring the total DNA concentration) indicating LNP transfer and second, LNP integrity (via comparing the measured DNA concentration encapsulated within LNPs to the total DNA concentration present in the sample) indicating disinfection, since LNPs disintegrate upon contact with disinfectants such as ethanol. The qPCR assay was carried out on a Roche Lightcycler 480 II and the protocol used was 6 min preactivation (95 °C) followed by 40 cycles of amplification (melting 4 s @ 95 °C, annealing 12 s @ 58 °C and elongation 6 s @ 72 °C). Samples were analyzed in duplicate with an average measurement difference of 0.09 ± 0.07 Ct. The negative control samples did not amplify at all, consequently LOD was set as Ct = 40.

### Fluorescent lotion and sampling

The fluorescent lotion (A) was prepared by mixing 100 µl of 2’-7’Dichlorofluorescin (5 g/l in milliQ water, Fluka-Chemie AG) with 300 µl milliQ, 300 µl glycerine (100%) and 600 µl sucrose (saturated solution in milliQ water). Per contamination spot 100 µl fluorescent lotion was applied using a pipette. To evaluate whether a surfaces were contaminated with fluorescent lotion, the surfaces were photographed under UV light (385 nm) using a smartphone camera. A second fluorescent lotion (B) was prepared by mixing 100 µl of a commercial fluorescent marker sold for contamination simulation (Detect + UV blue spray, FluoTechnik) with 300 µl milliQ, 300 µl glycerine (100%) and 600 µl sucrose (saturated solution in milliQ water). 100 µl fluorescent lotion was applied to a surface using a pipette. To evaluate whether surfaces were contaminated with fluorescent lotion, the surfaces were photographed under UV light (365 nm, K.LUV.HP.365 + LUN FluoTechnik) using a Sony DSC-RX10M2 camera (1/25 s, f/4.0; ISO 640).

### Pre-experiments

In order to test the limit of detection of LNP and fluorescent lotion, 100 µl of either LNP or fluorescent lotion liquid (A) or (B) was deposited on separate clean glass slides (cover slips, borosilicate glass, Roth) using a pipette. For both samples, the wetted glass surface was touched with a gloved fingertip (nitrile gloves, Sempercare) and the liquid was transferred to the surface of a second, clean glass slide by touching it with the same fingertip. This procedure was repeated in sequence for a total of 10 times, using a new glove for every transfer. The resulting two sets of 10 glass slides, together with a clean glass slide serving as a negative control, were analysed, for either LNP or fluorescent lotion contamination as described above.

To test the susceptibility towards ethanolic disinfection 100 µl of either LNPs or fluorescent lotion (A) were applied to the thenar of a gloved right hand (nitrile gloves, Sempercare). To establish the zero sample a clean glass slide was touched with the contaminated thenar. Then hands were rubbed together following the 3-step hand rub technique [14] for 15 s either using a palmful of milliQ water or ethanolic disinfectant (Sterillium med, 85 g ethanol / 100 g, Hartmann Group, Heidenheim). After rubbing, the gloves were sampled by touching another clean glass slide with the thenar. Because the doffing protocol requires disinfection of the same gloves five times, see Table A1, this procedure was repeated five times, wearing the same gloves, resulting in five samples taken. Then the contaminated glass slides were sampled for either LNP or fluorescent lotion as described above.

To establish a positive control for potential transfer events of LNPs on the different materials of the PPE, the transfers for intact and disinfected LNPs to and from different materials used in PPE of the Cantonal Hospital Baden were tested and quantified. Moreover, the sampling from such materials was evaluated. To be able to compare the results of transferring and sampling from different materials a clean glass slide was also tested to serve as a benchmark. For intact LNPs, 10 µl of LNP formulation was deposited on the material using a pipette and the spot was touched with a gloved fingertip. Afterwards, the glass surface representing the sampling of the material, and the fingertip, which conversely represents the transfer from the material, were sampled and analysed as described above. For disinfected LNPs, 100 µl of LNP formulation was deposited on the thenar of a gloved hand. Then, a palmful of disinfectant (Sterillium med, 85 g ethanol / 100 g, Hartmann Group, Heidenheim) was used to perform hand hygiene following the 3-step hand rub technique [14] for 15 s. Subsequently, the different materials were touched with gloved fingertips depositing disinfected LNPs on the material. Afterwards, the contaminated material was touched with a clean, gloved fingertip. The material and the fingertip were sampled and analysed as described above.

### Field study in Cantonal Hospital Baden

**Fig. 1:**
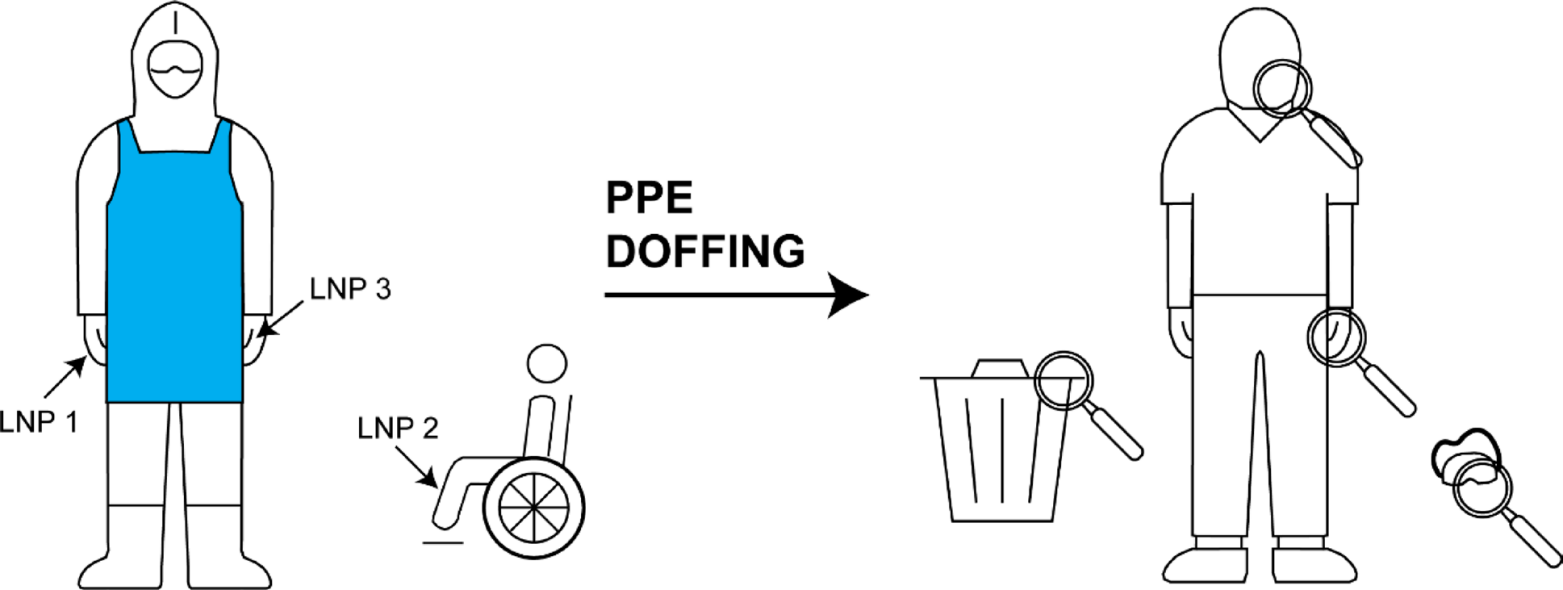
Schematic representation of the methodical procedure during the field-study showing the three deposition points indicated by arrows and the four sampling points indicated by magnifying glasses

The field study investigating the PPE doffing procedure for strict isolation at the cantonal hospital Baden was performed using LNPs. Before depositing any LNPs, background samples were taken from the surfaces that were to be contaminated. To evaluate the PPE doffing procedure different sites were contaminated using different LNP barcodes, see Fig. 1. The participants, infection control specialists from Cantonal Hospital Baden, donned PPE according to the protocol “donning and doffing of PPE for strict isolation” using an ethanol-based (80v%) disinfectant (see Table A1). The donning of the PPE was executed in the negative-pressure airlock. Next the participants proceeded to the patient room (high-risk area) and transferred a manikin, serving as a patient model, from a wheelchair to the floor and back into the wheelchair. A total of three runs were performed, where different sites were contaminated with LNPs. As soon as PPE was put on, but before interaction with the manikin, the LNP contaminations were placed on the PPE or other objects, as indicated in Fig. 1. The outer nitrile gloves were contaminated in run 1. In run 2 the outer nitrile gloves and the manikin were contaminated, and in run 3 the outer nitrile gloves, the manikin and the inner gloves were contaminated with LNPs. For each contamination site a different LNP barcode was used in order to distinguish different sources of contamination. Participants then relocated to the decontamination airlock and doffed PPE according to protocol. A floor plan indicating the different areas is shown in Figure A1 and the doffing procedure is summarized in Table A1. After complete removal of the PPE, meaning after completion of step 28 in Table A1, the rim of the waste bin in the doffing airlock, the manikin in the high-risk zone, the safety goggles worn, the hands and face (cheeks and nose) of each participant were sampled, see Fig. 1. Samples were taken from the participants in the anteroom. In each run two participants were donning and doffing simultaneously, totalling 6 sets of samples. All samples were tested for all three LNP barcodes used.

To avoid cross-contamination during deposition and sampling of the LNPs, the sampling team distributed the tasks so that one person was taking the background samples and another person was depositing the different LNP barcodes and taking the samples after doffing. After all runs one sample was taken from a known uncontaminated surface by the person who deposited the LNP barcodes, in order to establish a negative control for cross-contaminations.

## Results

### Pre-experiments

Two possible methods for investigating PPE doffing procedures, namely fluorescent lotion and lipid nanoparticles, were tested in pre-experiments to evaluate their suitability as viral surrogates in terms of limit of detection and sensitivity to disinfection.

**Fig. 2:**
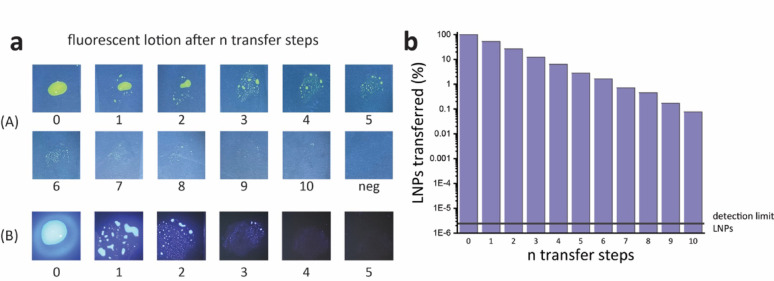
**a** Photographs of two different fluorescent lotions (**A**) and (**B**) after n transfer steps under UV irradiation.** b** LNPs transferred (%) after n transfer steps measured via qPCR with non-template negative control given as the detection limit of LNPs

After 10 consecutive transfer steps of fluorescent lotion (A), the contaminated surface was indistinguishable from the negative control, see Fig. 2a. The performance of a second fluorescent lotion (B), prepared from a commercial hygiene detection product was indistinguishable from the negative control after 4 consecutive transfer steps. In contrast, LNPs are still detectable after 10 transfer steps, see Fig. 2b, and the signal is still four orders of magnitude away from the detection limit of this method (= negative control).

**Fig. 3:**
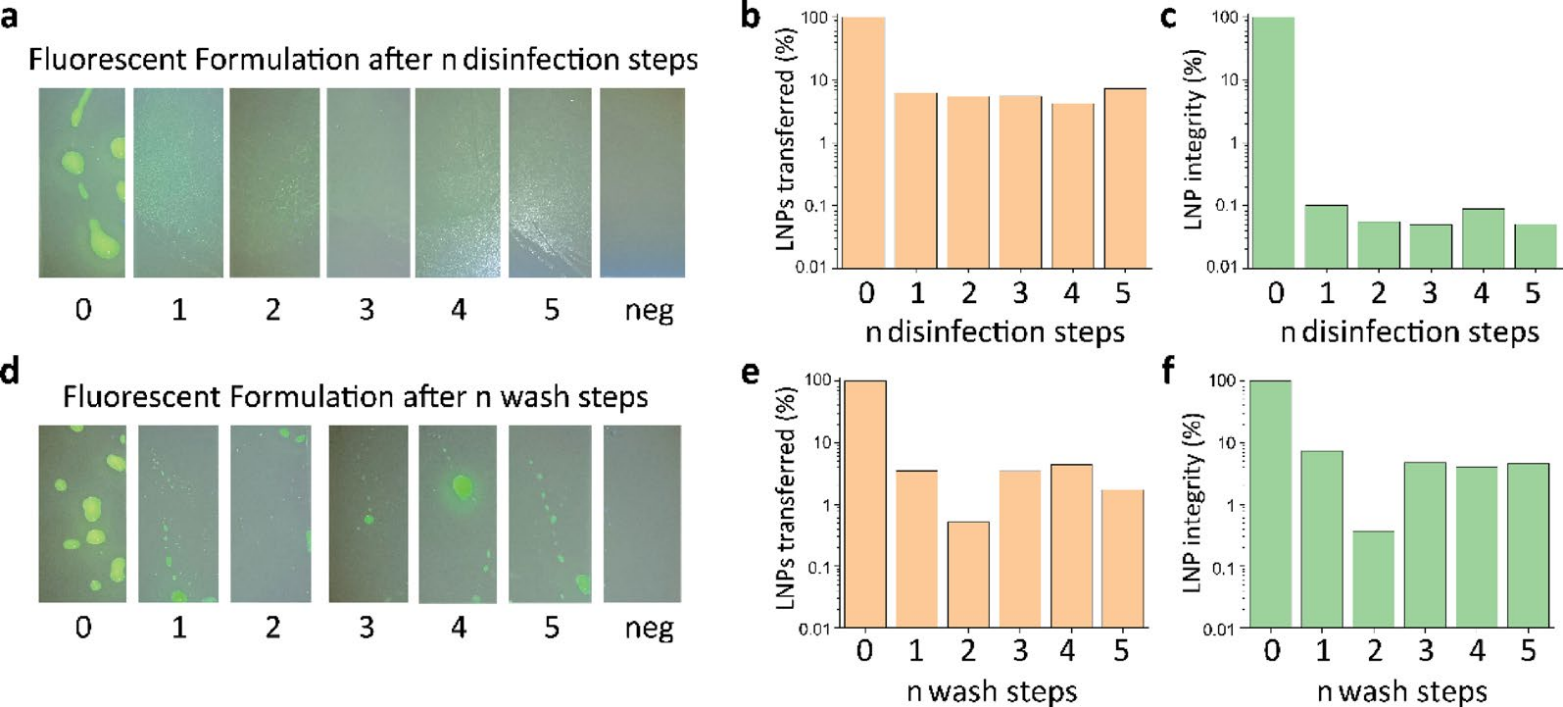
**a** Photographs of fluorescent lotion after n disinfection steps with ethanol based disinfectant with a clean glass surface as negative control **b** LNPs transferred (%) after n disinfection steps **c** LNP integrity (%) after n disinfection steps **d** Photographs of fluorescent lotion after n wash steps with water with a clean glass as negative control **e** LNPs transferred (%) after n wash steps **f** LNP integrity (%) after n wash steps

After hand hygiene the amount of fluorescent lotion and of LNPs decreased, regardless whether ethanolic disinfectant or water was used, see Fig. 3. Looking at LNP integrity, the integrity drops from 100% before disinfection to below 0.1% integrity after the first disinfection step, see Fig. 3c. When hands were rubbed using water instead of disinfectant, LNP integrity dropped to ca. 10%, see Fig. 3f. LNPs are efficiently transferred, regardless of whether hands were rubbed with disinfectant or with water, see Fig. 3b, 3e. Fluorescent lotion is efficiently transferred as well, however without the ability to quantify the transfer efficiency. Moreover, reduction in the amount of dye is observed, regardless of whether water or disinfectant was used, see Fig. 3a, d.

**Fig. 4:**
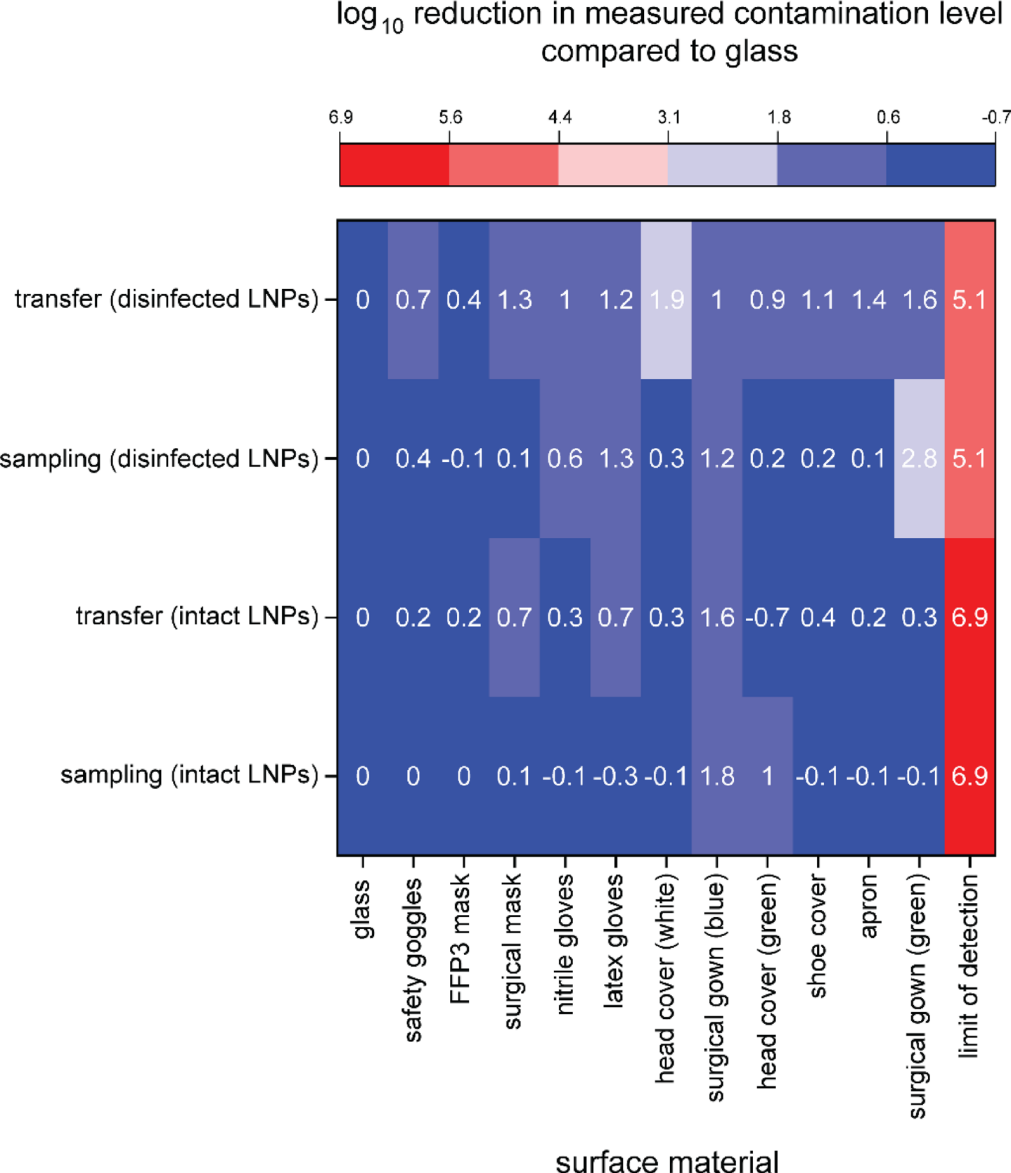
Heat map of sampling and transfer of intact and disinfected LNPs from different materials used in PPE compared to sampling and transfer from glass in log_10_ reduction. Analytical limit of detection, meaning non-template negative control, of the qPCR method is displayed in red

To establish a positive control for potential transfer events of LNPs on the different materials of the PPE, Fig. 4 shows that LNPs, intact or disinfected, can be efficiently sampled and transferred from all materials of the PPE. The measured levels of contamination, meaning the measured LNP concentrations, are orders of magnitudes away from the detection limit of the LNPs using qPCR (distance from negative control, red in Fig. 4).

### Field study in Cantonal Hospital Baden

**Fig. 5:**
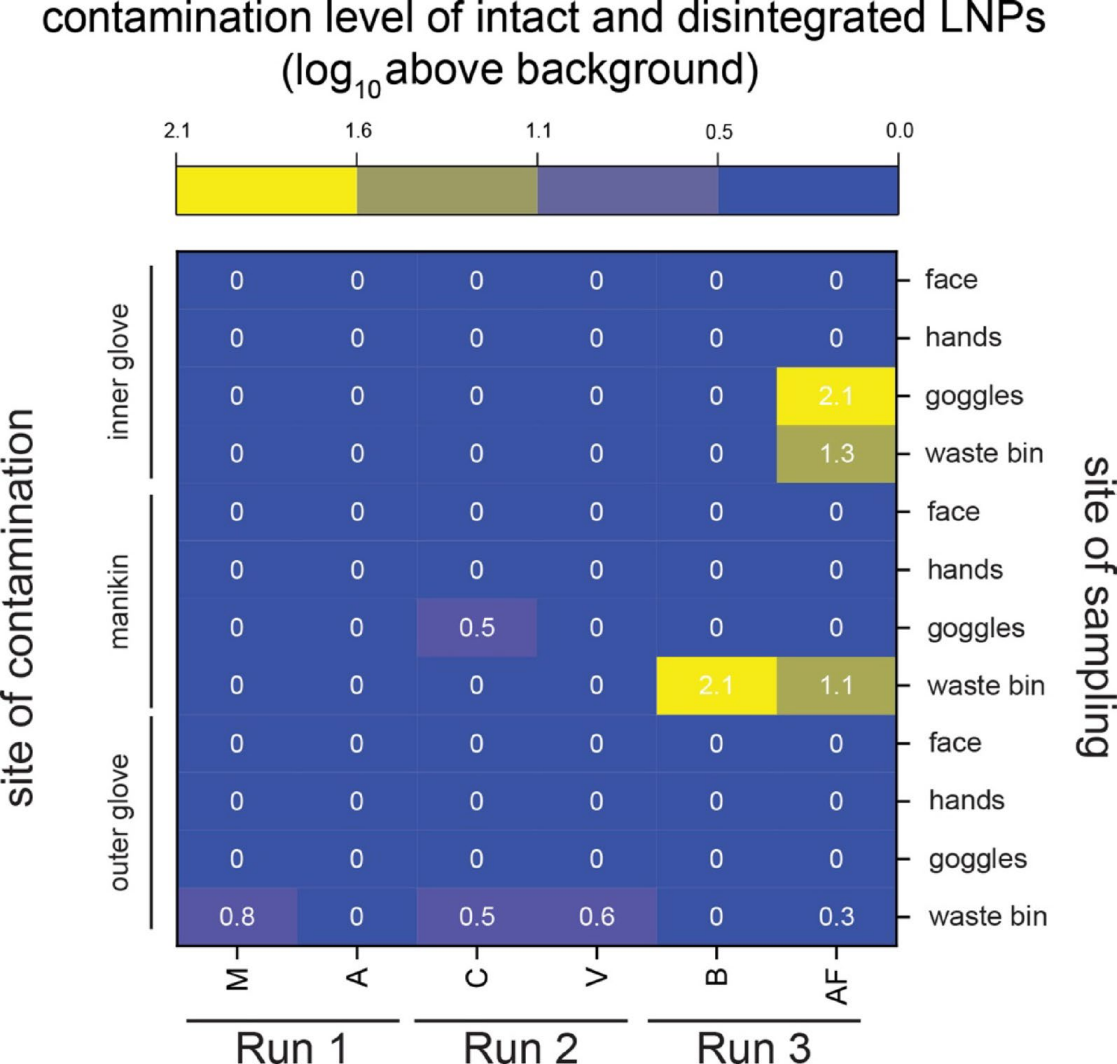
Heatmap of the measured contamination level originating from overall LNP concentration (intact and disintegrated) in the samples taken from face, hands, goggles and waste bin for each participant. The lowest four rows show the contamination levels originating from the outer glove, the middle four rows show the contamination levels originating from the manikin and the four uppermost rows show contamination originating from the inner gloves. The contamination levels are expressed in log_10_ above background

Figure 5 shows the total contamination level of the respective sites sampled, represented by the LNP concentration measured. Several sites were found positive, meaning a LNP concentration was measured that was higher than the measured background concentration. The waste bin was found positive for the barcode deposited on the HCW's outer glove in four out of six samples, with a level of contamination of ca. 0.5 log_10_ over the background. In addition, the rim of the waste bin was found positive twice for the barcode applied to the manikin, with a level of contamination of 2.1 log_10_ above background, and once for the barcode from the inner gloves. The safety goggles were never found positive for the contamination originating from the outer gloves. However, they were once found positive for the barcode deposited on the manikin and once positive for the barcode deposited on the inner gloves, with a measured level of contamination of 2.1 log_10_. The hands and faces of participants were never found positive for any of the barcodes deposited.

**Fig. 6:**
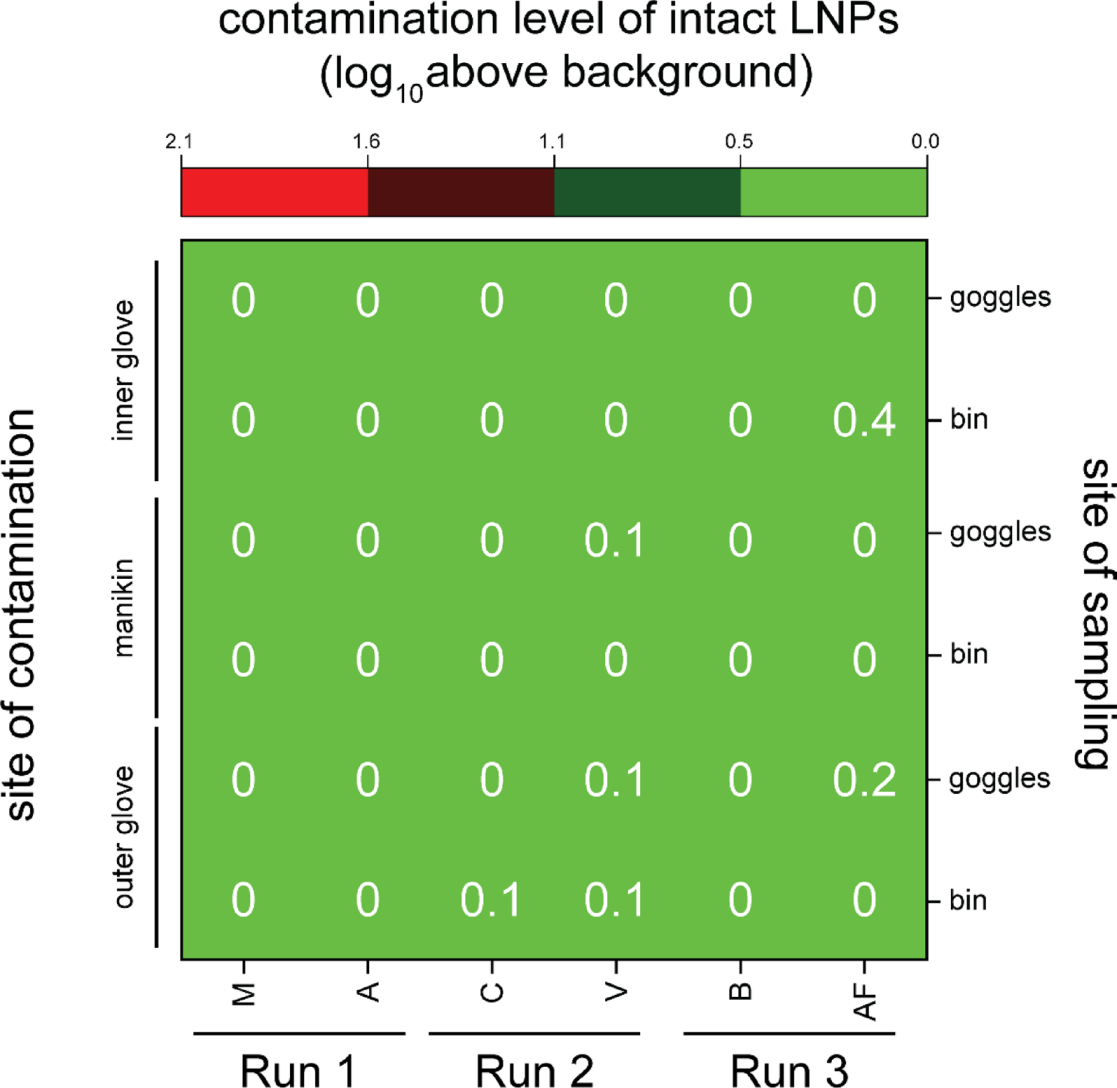
Heatmap of the measured contamination level originating from intact LNPs in the samples taken from goggles and waste bin for each participant. The lowest two rows show the contamination levels originating from the outer glove, the middle two rows show the contamination levels originating from the manikin and the two uppermost rows show contamination originating from the inner gloves. The contamination levels of intact LNPs are expressed in log_10_ above background

Additionally, LNP integrity in each sample taken from the waste bin and the goggles was measured and the portion of the sample consisting of intact LNPs is displayed in Fig. 6.

A fraction (0.1 – 0.4 log_10_) of the contamination level found originated from intact LNPs, see Fig. 6. This shows that the LNPs found were treated with disinfectant during the doffing procedure, which contrasts with the highest contamination level measured for overall (intact + disinfected) LNP contamination (2.1 log_10_, Fig. 5).

## Discussion

The goal of this study was to evaluate LNPs for their suitability as viral surrogates to investigate PPE doffing procedures. For this, LNPs were tested in pre-experiments, and compared to two fluorescent lotions.

The pre-experiments showed that: First, LNPs have a lower detection limit compared to fluorescent lotions, see Fig. 2. This detection limit is especially important when modelling pathogens, which are still infectious at very low doses, such as Ebola, which has an infectious dose as low as 10 viruses [13], and can be detected at concentrations below 1 copy/µl using PCR based assays. The state-of-the-art method, fluorescent lotion, has a detection limit that is far too high, which could lead to incorrect results and a false sense of security. Second, both LNPs and fluorescent lotion are efficiently transferred from gloved hands after disinfection, see Fig. 3. Due to the difficulty in precisely reproducing the hand wash procedure the data showed some noise, when water was used instead of ethanol (see Fig. 3e, f). Nonetheless, only LNPs can be used to determine if a contamination has been treated with a disinfectant, see Fig. 3c. Therefore, the use of LNPs enables the measuring of two parameters via qPCR: First, overall LNP transfer indicating contamination, Second, LNP integrity indicating if the contamination was treated by a disinfectant. In contrast, fluorescent lotion does not indicate whether a disinfectant has been used and transfer quantification can only be approximated by examining the photographs taken. Consequently, LNPs were found to possess more suitable properties to act as viral surrogates than fluorescent lotions and were, therefore, subsequently used in the field study to investigate the PPE doffing procedure for strict isolation of a patient at Cantonal Hospital Baden.

In investigating the PPE doffing in the field study, samples taken from participants’ faces and hands after doffing were never found positive for any of the LNP barcodes deposited. This shows that the doffing procedure can be regarded as effective, since no surrogates were found on the skin of HCWs. This means that even at the very low detection limits offered by qPCR no transfer or contamination of the LNPs had taken place. This result is especially reliable and informative, since the procedure was tested six times with different HCWs performing the doffing and, since the detection limit of the analysis methods is extremely low (LOD < 1 LNP), even more so when compared to the detection limit of the fluorescent lotions.

Another result of the field study was that the waste bin was found positive for all LNP barcodes deposited, see Fig. 5. This indicates that LNPs were transferred to the waste bin via different pathways. However, in all samples taken, the LNPs were found to have been treated by the disinfectant, see Fig. 6, with only an extremely small fraction of intact LNPs (0.003%). This demonstrates that LNPs are a useful and sensitive tool to detect ethanolic disinfection. However, the LNPs serve as a mere model for viral contamination and a given virus may not have the same susceptibility towards the disinfectant as LNPs have. Viruses show different levels of susceptibility towards hand hygiene, and for most cases the susceptibility of a virus of interest to ethanolic disinfecting solutions has been investigated. [13, 15–18] Nevertheless, the value of the detection of disinfection via LNPs is high, since it is possible indicate that all environmental contaminations have been effectively treated with disinfectant. This study was focused on analysing HCW self-contamination during doffing, however in future studies the LNPs could also be used to elucidate the sources of contamination from the HCW environment to the PPE. As already noted, the LNPs don't prove if a given virus would have been made inactive during the disinfection procedure, but they can be used if ethanolic disinfection steps were performed. We anticipate that future adaptions of the LNPs will also allow for the detection of other disinfection procedures, which would also affect more resilient pathogens, such as those also affecting non-enveloped viruses.

In general, the safety of the PPE doffing procedure is two-fold. The first layer of safety is the inhibition of transfer to key surfaces (HCW’s hands and faces) and the second layer is the inactivation of potential contaminants through disinfection to render the inevitable transfer to environmental surfaces (waste bin, goggles) non-infectious. These two layers of safety can be measured and quantified using LNPs: transfer to key surfaces is detected via overall LNP contamination at very high sensitivities, and disinfection is detected via LNP integrity. These distinctions cannot be made using fluorescent lotions, as their detection limits are too high to reliably investigate the first layer (transfer to key surfaces), and no detection of the use of ethanol based disinfectants in the second layer is possible.

## Conclusion

In conclusion, the safety of PPE doffing procedures relies on a dual-layered approach: First, minimizing the transfer of contaminants to critical surfaces such as the HCWs’ hands and faces, and Second, ensuring that any contaminants transferred to environmental surfaces are rendered non-infectious through effective disinfection. Lipid nanoparticles (LNPs) offer a novel, highly sensitive tool to evaluate both these layers, detecting minute levels of contamination and assessing disinfection effectiveness via nanoparticle integrity. Such capabilities are not available with traditional fluorescent lotion methods. This study marks the first use of LNPs in evaluating PPE doffing protocols, demonstrating their potential as harmless viral surrogates with a very low detection limit. Results confirmed that the PPE doffing protocol for strict isolation in place at the Cantonal Hospital Baden prevents contamination of HCWs’ hands and faces (n = 6), but reveals environmental surfaces as sites of contamination. Though LNPs cannot confirm actual viral inactivation, they offer a substantial improvement over existing methods, helping to identify blind spots and improve the overall safety of PPE doffing procedures.

## Supplementary Information

Below is the link to the electronic supplementary material.

Supplementary Material 1

## Data Availability

The authors declare that the data supporting the findings of this study are available within the paper and its Supplementary Information files. Should any raw data files be needed in another format they are available from the corresponding author upon reasonable request.

## Abbreviations

HCW: Health care worker
LNP: Lipid nanoparticles encapsulating DNA
PPE: Personal protective equipment
qPCR: Quantitative polymerase chain reaction
