# Supplementary material for: Doffing procedures of personal protective equipment evaluated with lipid nanoparticles as viral surrogates: uncovering potential blind spots
Source: Antimicrob Resist Infect Control. 2025 Dec 11;15:7. doi: 10.1186/s13756-025-01680-w (PMC12801874; doi:10.1186/s13756-025-01680-w)
Supplement: Supplementary file 1 — Supplementary Material 1 [file 13756_2025_1680_MOESM1_ESM.docx]

# Supplemental Material for Doffing procedures of personal protective equipment evaluated with lipid nanoparticles as viral surrogates – uncovering potential blind spots

Authors: Lara Pfuderer^1^, Andree Friedl MD^2^, Benedikt Wiggli MD^2^, Robert Grass PhD^1^*

(1) Institute for Chemical- and Bioengineering, Department of Chemistry and Applied Biosciences, ETH Zurich

(2) Department of Internal Medicine, Cantonal Hospital of Baden, Baden, Switzerland

Corresponding author: * Prof Dr Robert Grass, Vladimir-Prelog-Weg 1-5/10, 8093 Zürich, Switzerland, +41 44 633 63 34, robert.grass@chem.ethz.ch

- **Table A1**: Summary of doffing protocol from KSB for isolating a high-risk patient.
- **Figure A1**: Floor plan of the facilities used

**Table A1**: Summary of doffing protocol from KSB for isolating a high-risk patient. Detailed protocol is available on request at the division of infectious disease at cantonal hospital Baden.

| **Step** | **Action** |
| --- | --- |
| 1 | Disinfect outer gloves |
| 2 | Move from patient room into doffing airlock |
| 3 | Disinfect outer gloves |
| 4 | Remove plastic apron and dispose it in the bin |
| 5 | Disinfect outer gloves |
| 6 | Sit on stool and remove shoe covers |
| 7 | Disinfect outer gloves |
| 8 | Disinfect stool |
| 9 | Disinfect outer gloves |
| 10 | Remove outer gloves and dispose it in the bin |
| 11 | Disinfect inner gloves |
| 12 | Remove goggles and put them on the floor |
| 13 | Disinfect inner gloves |
| 14 | Remove head cover and dispose it in the bin |
| 15 | Disinfect inner gloves |
| 16 | Remove apron and dispose it in the bin |
| 17 | Disinfect inner gloves |
| 18 | Remove inner gloves and dispose it in the bin |
| 19 | Disinfect hands |
| 20 | Put on a new pair of gloves |
| 21 | Remove surgical face mask and dispose it in the bin |
| 22 | Disinfect new gloves |
| 23 | Remove FFP3 mask and dispose it in the bin |
| 24 | Disinfect new gloves |
| 25 | Remove boots |
| 26 | Disinfect new gloves |
| 27 | Remove gloves and dispose it in the bin |
| 28 | Disinfect hands and move from doffing airlock into anteroom |


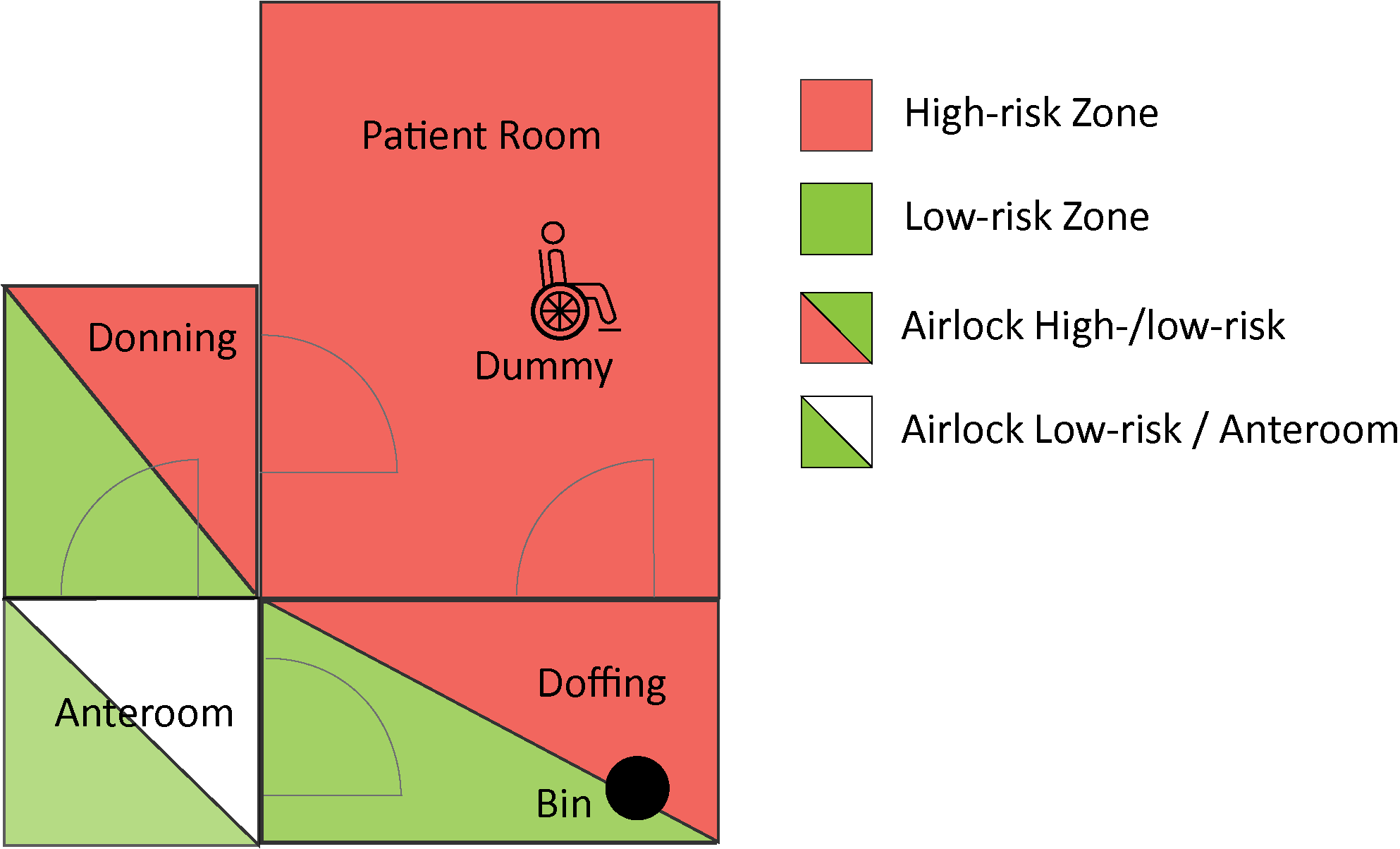


**Figure A1**: Floor plan of the facilities used to indicate the different risk areas and the airlocks where donning and doffing was performed, respectively.
